# Preservation of Sexual Function 5 Years After Water Vapor Thermal Therapy for Benign Prostatic Hyperplasia

**DOI:** 10.1016/j.esxm.2021.100454

**Published:** 2021-10-30

**Authors:** Kevin T. McVary, Ahmad El-Arabi, Claus Roehrborn

**Affiliations:** 1 Stritch School of Medicine, Loyola University Medical Center, Maywood, IL, USA; 2 UT Southwestern Medical Center, Urology, Dallas, TX, USA

**Keywords:** Benign Prostatic Hyperplasia, Sexual Dysfunction, Erectile Dysfunction, Ejaculatory Dysfunction, Lower Urinary Tract Symptoms, Water Vapor Thermal Therapy

## Abstract

**Background:**

Erectile dysfunction (ED) and ejaculatory dysfunction (EjD) are known outcomes of traditional surgery and some pharmacotherapies for treatment of benign prostatic hyperplasia (BPH). Minimally invasive treatment options, including water vapor thermal therapy (WVTT), are now available to treat lower urinary tract symptoms (LUTS) due to BPH.

**Aim:**

The objective of this analysis was to evaluate long-term impact of a single water vapor thermal therapy procedure on erectile and ejaculatory function in subjects enrolled in the Rezum II prospective, multicenter, randomized, blinded controlled trial.

**Methods:**

Fifteen centers enrolled 197 subjects with International Prostate Symptom Score (IPSS) ≥ 13, maximum flow rate (Qmax) ≤ 15 mL/s, and prostate volume 30–80 cc. Subjects were randomized (2:1) to (WVTT) or sham procedure (control) and followed for 5 years. Erectile and ejaculatory functions were quantitatively assessed at baseline and yearly thereafter. After 3 months, control subjects could opt to requalify for cross-over to WVTT and were followed for 5 years. Results of the per protocol analysis were reported previously. The current post hoc analysis was performed on all treated subjects who were sexually active at baseline with no other surgical or medical management for BPH during the 5-year study period.

**Outcomes:**

LUTS was evaluated using IPSS, Benign Prostatic Hyperplasia Impact Index (BPHII), and Qmax. Sexual function was assessed using the International Index of Erectile Function (IIEF-EF) and Male Sexual Health Questionnaire for Ejaculatory Dysfunction (MSHQ-EjD).

**Results:**

A total of 197 subjects (136 treated, 61 control) were enrolled in the study, and 53 control subjects opted to cross-over and receive WVTT. All subgroups experienced significant, durable improvement in IPSS (*P* < .0001). Subjects with normal sexual function at baseline had little change in function over 5 years (IIEF-EF: −2.4 ± 8.9, *P* = .1414; MSHQ-EjD Function: −1.6 ± 3.2, *P* = .0083; MSHQ-EjD Bother: −0.5 ± 1.6, *P* = .1107). Subjects with baseline medical history of ED and EjD showed slight decline over time that was not clinically significant (ED, IIEF-EF: −3.0 ± 10.1, *P* = .1259; MSHQ EjD Function: −2.3 ± 4.7, *P* = .0158; MSHQ-EjD Bother: −0.1 ± 2.6, *P* = .7764; EjD, IIEF-EF: −4.1 ± 9.2, *P* = .0127; MSHQ EjD Function: −1.6 ± 4.8, *P* = .1970; MSHQ-EjD Bother: −0.4 ± 2.6, *P* = .440).

**Clinical Implications:**

Treatment for BPH with Rezum durably improved IPSS without clinically significant impact on sexual function. Patients with baseline ED/EjD may expect continued decline from other causes but are unimpacted by the therapy.

**Strengths & Limitations, Conclusion:**

The results are limited by the post-hoc nature of the analysis and attrition over the 5-year follow-up but provide long-term evidence of durable outcomes after treatment with Rezum without impact on sexual function scores. **McVary KT, El-Arabi A, Roehrborn C. Preservation of Sexual Function 5 Years After Water Vapor Thermal Therapy for Benign Prostatic Hyperplasia. Sex Med 2021;9:100454.**

## INTRODUCTION

Benign prostatic hyperplasia (BPH) is a histological condition that may progress to prostate enlargement. Along with lower urinary tract symptoms (LUTS), worsening of sexual function is a common occurrence in men with LUTS/BPH.^1^^,^^2^^,^^3^ LUTS is an independent risk factor for sexual dysfunction, and about 50–60% of men with LUTS have co-existing erectile dysfunction (ED).^1^^,^^4^ In addition to this independent risk factor, men who seek treatment for LUTS/BPH are often prescribed α-blockers and 5-α reductase inhibitors as a first-line treatment. These medications can have a negative impact on ED and ejaculatory (EjD) function and sexual quality of life (QoL).^5^^,^^6^ Alternatively, surgical modalities, although considered the definitive treatment of LUTS/BPH, have been associated with sexual side effects, including retrograde ejaculation, and ED.^7^

Although transurethral resection of the prostate (TURP) is still considered the gold-standard for surgical treatment of LUTS/BPH, ED is a reported complication of this procedure.^8^^,^^9^ Minimally invasive options are now available for the management of LUTS/BPH and are shown to cause fewer de novo sexual side effects.^7^^,^^10^ Among the newest minimally invasive options is transurethral water vapor thermal therapy (Rezūm, Boston Scientific Company Inc, Marlborough, MA) which utilizes convective radiofrequency water vapor to ablate prostate tissue. American Urological Association (AUA) BPH Clinical Guidelines support the use of water vapor thermal therapy in patients with prostate volume less than 80 g. Since published data report no de novo ED after this treatment, the AUA guidelines support offering water vapor thermal therapy to patients who desire preservation of erectile and ejaculatory dysfunction.^10^^,^^11^

A randomized clinical trial of Rezum compared with a sham procedure found significant and durable improvement in LUTS with preserved erectile and ejaculatory function through 5-year follow-up.^12^^,^^11^^,^^13^^,^^14^^,^^15^ The goal of the current analysis is to provide 5 year sexual function outcome data in a subpopulation of this study who were sexually active at baseline regardless of their overall level of erectile function. Specific attention is paid to those who had ED/EjD at baseline compared with those who had normal baseline sexual function. We hypothesize that water vapor thermal therapy does not impact sexual function in males treated for BPH.

## MATERIALS AND METHODS

The Rezum II pivotal study was conducted at 15 centers in the United States with a follow-up period of 5 years. Approval of the protocol was granted by an institutional review board for participating investigational sites, and participants signed written informed consent form prior to participation. Key inclusion criteria included age ≥50 years, International Prostate Symptom Score (IPSS) ≥13, maximum flow rate (Qmax) ≤ 15 mL/s, prostate volume of 30–80 cm^3^ without restrictions on the presence of a middle lobe. Eligible subjects also had no prior prostate intervention or surgery of the prostate, and underwent a washout period for antihistamines, α-blockers, anticholinergics, daily dose PDE-5 inhibitors, 5-α reductase inhibitors, estrogen, androgen suppressing drugs, and anabolic steroids. The specific methodology and per protocol analysis of this study have been previously published.^12^^,^^11^^,^^14^^,^^15^^,^^16^

A total of 197 subjects were randomized to treatment and control in a 2:1 ratio using a permuted-block randomization schedule with varying block size, stratified on center and baseline IPSS. Unblinded at the 3-month follow-up visit, 53 of 61 control subjects requalified based on IPSS and Qmax eligibility criteria and elected to receive Rezum thermal therapy. The other eight control group subjects had Qmax scores that were too high (N = 3), IPSS scores that were too low (N = 1), opted to use an exclusionary medication (N = 2) or elected to not crossover (N = 2). Crossover treatment occurred within 3–6 months postenrollment date. Subjects in the treatment and crossover arms were followed at 3, 6, and 12 months postwater vapor thermal therapy, and then annually until 5 years.

The current post hoc analysis was performed on a subset of Rezum-treated subjects who were sexually active at baseline with no other surgical or medical management for LUTS/BPH during the 5-year study period and includes those who had a baseline medical history of ED or EjD and those who had normal baseline function. Baseline sexual activity was assessed by an answer of 1–2 or greater attempts in response to the question, “over the past four weeks, how many times have you attempted sexual intercourse?” A response of “no attempts” was considered as inactive. ED was defined based on reported history at baseline. EjD was defined based on reported history of decreased volume of ejaculate and was not confirmed by postejaculate urine sample.

Patient-reported outcomes of ED and EjD were assessed at all time points with standardized and 2 validated questionnaires. The MSHQ-EjD is used to evaluate EjD and has two domains, function and bother.^16^ The IIEF-EF is the gold standard measure of male sexual function, and of the five domains assessed, this study focused on erectile function.^17^

### Statistical Methods

The follow-up values and changes from baseline were summarized and reported as mean ± standard deviation. Missing data were not imputed. Evaluation of whether the mean change from baseline is nonzero was performed using the paired *t*-test. Two-sided *P* values are reported without adjustment for multiple comparisons, and *P* < .05 was considered statistically significant. Analyses were conducted using SAS Software, version 9.4 (SAS Institute Inc, Cary, NC).

## RESULTS

A total of 125 subjects from the treatment and cross-over groups were included in this subanalysis with 67 subjects remaining at the 5-year follow-up. Study attrition was summarized in detail in a prior manuscript on the main study findings.^10^ Table 1 includes the baseline characteristics of the entire population of sexually active men who are included in this analysis. For this population, IPSS and IPSS-QoL scores improved significantly, starting at the 3-month visit and were consistently sustained through 5 years (*P* < .0001; Supplemental Table). Similarly, Qmax and BPHII improved significantly from baseline and the statistically significant improvement was seen at all timepoints through 5 years (*P* < .0001).

**Table 1:** Baseline characteristics of sexually active subgroup

Characteristic	Mean ± SD (n = 125)
Age at screening, years	61.2 ± 6.6
PSA, ng/mL	2.0 ± 1.4
Prostate Volume, cm^3^	43.9 ± 11.7
PVR, mL	86.8 ± 61.2

PSA = prostate-specific antigen; PVR, postvoid residual.

**Figure 1:**
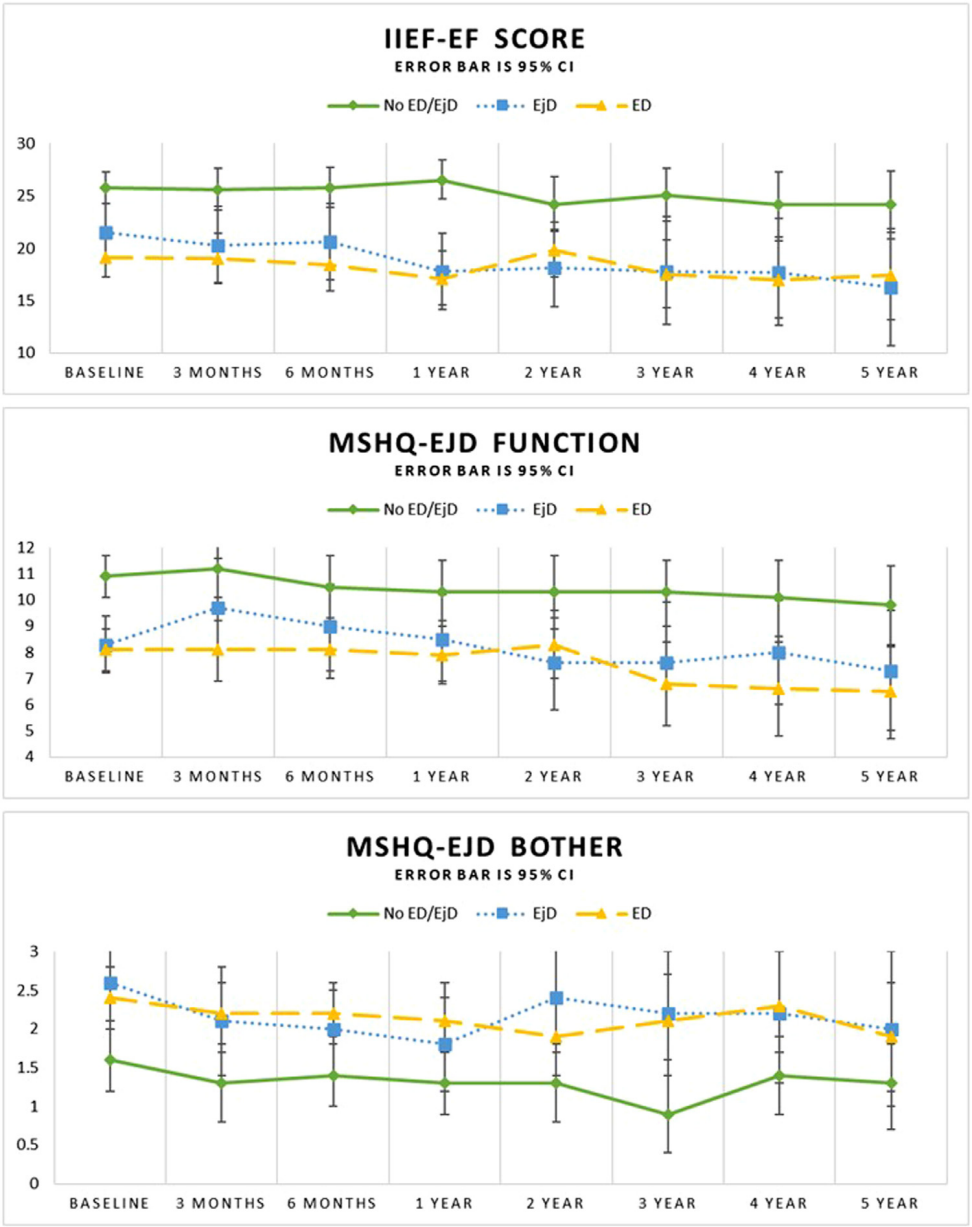
Change in sexual function scores by baseline medical history.

**Figure 2:**
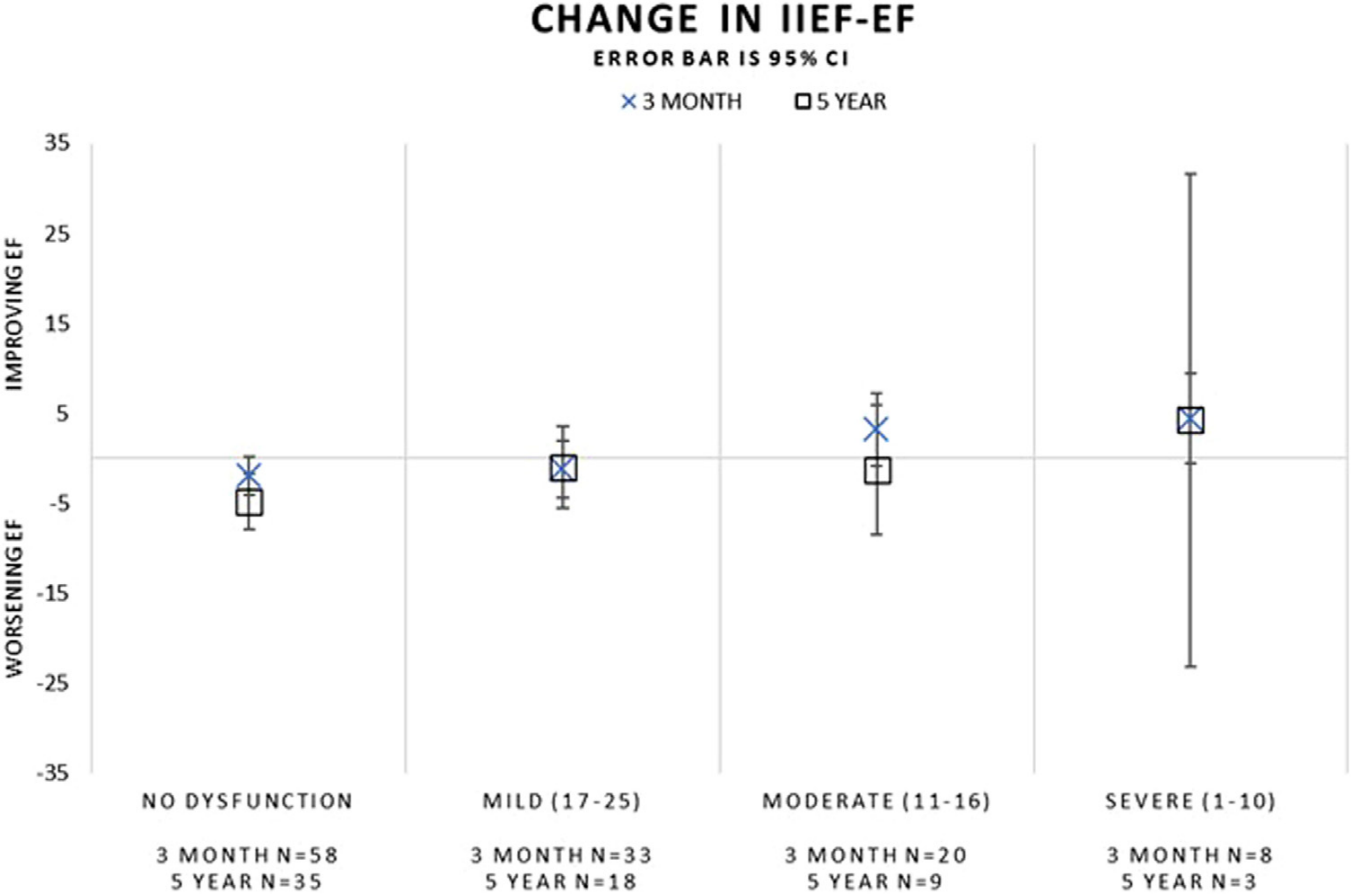
Change in sexual function by baseline IIEF-EF scores.

Subgroup analysis evaluated these outcomes based on the baseline status, including those without ED/EjD at baseline, and those with ED and/or EjD at baseline (Table 2 and Figure 1). Subjects reporting both ED and EjD at baseline are represented in the ED and EjD analyses. Improved IPSS scores were observed at all timepoints in subjects without baseline ED/EjD (*P* < .0001) as well as those with baseline ED/EjD (*P* < .0001). Subjects without ED/EjD at baseline had an absolute change in IIEF-EF of −2.4 ± 8.9 (*P* = .1414) over 5 years of follow-up compared to those with baseline ED (−3.0 ± 10.1, *P* = .1259) or EjD (−4.1 ± 9.2, *P* = .0127). MSHQ-EjD function scores showed some change during the 5-year follow-up for the No ED or EjD and ED groups and no change for the EjD group (No ED or EjD: −1.6 ± 3.2, *P* = .0083; ED: −2.3 ± 4.7, *P* = .0158; EjD: −1.6 ± 4.8, *P* = .1970). MSHQ-EjD bother scores were not significantly different at 5 years post-treatment (No ED or EjD: −0.5 ± 1.6, *P* = .1107; ED: −0.1 ± 2.6, *P* = .7764; EjD: −0.4 ± 2.6, *P* = .4440; Table 2 and Figure 1). The change in IIEF-EF scores during follow-up were also evaluated considering the baseline IIEF-EF score clinical categories (no dysfunction, mild, moderate, severe). The change from baseline was similar at the 3-month and 5-year visits across all subgroups (Figure 2) and baseline IIEF-EF scores did not predict follow-up IIEF-EF scores.

**Table 2:** Change in sexual function by baseline medical history

Baseline status		3 months	6 months	1 year	2 years	3 years	4 years	5 years
IPSS⁎								
No ED or EjD	N (paired values)	53	52	48	40	37	36	33
	Change ± SD	−11.9 ± 6.5	−11.6 ± 7.1	−12.0 ± 6.6	−11.6 ± 7.5	−12.8 ± 5.8	−10.4 ± 6.7	−10.3 ± 7.7
	*P* value	<.0001	<.0001	<.0001	<.0001	<.0001	<.0001	<.0001
ED	N (paired values)	61	56	51	45	37	31	29
	Change ± SD	−11.1 ± 7.9	−12.3 ± 7.7	−11.9 ± 8.2	−10.5 ± 8.4	−11.6 ± 8.0	−11.8 ± 9.0	−11.5 ± 9.0
	*P* value	<.0001	<.0001	<.0001	<.0001	<.0001	<.0001	<.0001
EjD	N (paired values)	29	29	30	26	20	18	17
	Change ± SD	−12.6 ± 8.5	−13.3 ± 8.9	−10.8 ± 9.0	−9.3 ± 8.5	−11.8 ± 7.5	−10.1 ± 10.0	−11.2 ± 9.2
	*P* value	<.0001	<.0001	<.0001	<.0001	<.0001	<.0001	<.0001
IIEF-EF†								
No ED or EjD	N (paired values)	51	49	44	38	35	35	32
	Change ± SD	−0.5 ± 7.4	−0.7 ± 5.7	0.1 ± 6.0	−2.0 ± 7.4	−1.5 ± 8.0	−2.1 ± 8.5	−2.4 ± 8.9
	*P* value	.6125	.4018	.9202	.0988	.2795	.1514	.1414
ED	N (paired values)	60	55	50	45	37	30	28
	Change ± SD	−0.5 ± 8.6	−0.3 ± 7.7	−2.6 ± 9.5	0.1 ± 7.6	−1.9 ± 9.1	−3.0 ± 9.8	−3.0 ± 10.1
	*P* value	.6771	.7664	.0636	.9532	.2222	.1012	.1259
EjD	N (paired values)	29	28	30	25	19	18	17
	Change ± SD	−0.9 ± 7.9	−0.3 ± 5.9	−3.7 ± 8.3	−2.8 ± 8.4	−3.2 ± 9.7	−2.9 ± 9.0	−4.1 ± 9.2
	*P* value	.5443	.7988	.0206	.1031	.0724	.0552	.0127
MSHQ-EjD function‡								
No ED or EjD	N (paired values)	51	49	45	38	36	34	31
	Change ± SD	0.3 ± 4.3	−0.8 ± 3.9	−0.9 ± 3.2	−0.9 ± 4.0	−1.2 ± 3.4	−1.3 ± 3.8	−1.6 ± 3.2
	*P* value	.6724	.1892	.0600	.1534	.0357	.0483	.0083
ED	N (paired values)	61	54	50	44	37	29	28
	Change ± SD	−0.1 ± 4.1	0.0 ± 3.5	−0.3 ± 3.7	−0.1 ± 4.1	−1.5 ± 4.4	−2.2 ± 5.0	−2.3 ± 4.7
	*P* value	.8042	.9688	.5178	.8283	.0385	.0269	.0158
EjD	N (paired values)	29	27	30	25	19	17	16
	Change ± SD	1.4 ± 4.4	0.6 ± 4.1	0.2 ± 4.1	−0.8 ± 4.5	−1.3 ± 4.8	−0.8 ± 4.6	−1.6 ± 4.8
	*P* value	.0991	.4560	.7894	.3547	.2658	.4711	.1970
MSHQ-EjD Bother⁎								
No ED or EjD	N (paired values)	51	50	45	38	36	34	31
	Change ± SD	−0.3 ± 2.1	−0.2 ± 2.1	−0.4 ± 1.7	−0.5 ± 2.1	−0.8 ± 1.8	−0.3 ± 1.6	−0.5 ± 1.6
	*P* value	.3177	.3841	.1156	.2458	.0145	.4185	.1107
ED	N (paired values)	61	55	51	44	37	29	28
	Change ± SD	−0.1 ± 1.8	−0.2 ± 1.7	−0.2 ± 1.9	−0.4 ± 1.6	−0.0 ± 1.9	0.2 ± 2.2	−0.1 ± 2.6
	*P* value	.5320	.3413	.4241	.1038	.9328	.5561	.7764
EjD	N (paired values)	29	29	30	26	19	17	16
	Change ± SD	−0.5 ± 1.9	−0.7 ± 1.7	−0.8 ± 2.0	−0.2 ± 1.8	−0.1 ± 1.7	−0.3 ± 2.1	−0.4 ± 2.6
	*P* value	.1866	.0367	.0423	.5066	.7024	.3419	.4440

ED = erectile dysfunction; EjD = ejaculatory dysfunction.

*P* < .05 is considered to be statistically significant. Analysis population includes all treatment and crossover arm subjects that underwent treatment with Rezum, were sexually active at baseline, and did not have other medical treatments during the study period.

Percent change analyses do not include subjects with baseline values of zero.

⁎ Decrease indicates improvement.

† Increase indicates improvement.

‡ Decrease indicates a decline in function.

## DISCUSSION

The current report expands our understanding of the long-term effect of a single water vapor thermal therapy treatment on sexual function. In our analysis, sexually active subjects experienced a modest and gradual decline in sexual function during the 5-year follow-up. IIEF-EF scores demonstrated a statistically significant change at 4 and 5 years post-treatment but remained within the mild-moderate range throughout the study. A nonclinically significant decline in sexual function was observed among subjects in the baseline ED and EjD groups compared to subjects reporting no ED/EjD who showed little change. In subjects with pre-existing ED and/or EjD, LUTS improvement occurred similarly between those with baseline ED and EJD compared to those without baseline dysfunction.

Our results suggest that treatment of LUTS/BPH with water vapor thermal therapy does not have a clinically meaningful impact on erectile or ejaculatory function since patients stay within their score category. In light of other studies evaluating LUTS/BPH and sexual function, these results are reassuring. The causal relationship and pathophysiologic mechanism between LUTS/BPH and sexual dysfunction remains unclear.^16^ Despite studies investigating this association, prior analysis of data from the MTOPS randomized trial found that declining sexual function was not strongly associated with worsening LUTS in men assigned to placebo.^18^ Further, in an analysis of claims data, the baseline prevalence of BPH diagnosed in a population of men with ED was only 1.5% suggesting the frequent independence of ED symptoms from LUTS/BPH.^19^ In a population-based analysis of men 40 and over, nearly a quarter of men reported experiencing symptoms of ED, while only 5% reported a concurrent diagnosis of BPH.^20^

The gradual decline in sexual function over 5 years seen in this analysis possibly reflects the para-aging process which has been well-documented in other publications and reported to be around 30%.^21^ A review of the natural history of ED found that the prevalence of ED increased with age in all epidemiological studies reviewed.^22^ The prevalence and severity of erectile dysfunction in the Massachusetts Male Aging Study increased steadily from age 40 to 70, and was associated with coronary risk factors.^23^^,^^24^ Similar trends are seen in studies of prevalence of ED conducted worldwide. Importantly this decline in sexual function is associated with a decline in wellbeing thus it should not be ignored as a public health concern.^25^^,^^26^^,^^27^

Erectile function (IIEF-EF) scores in our study trended toward gradual decline throughout the study period starting 1 year after treatment, although based on the clinical categorical relevance, subjects remained in the dimension of mild to moderate dysfunction throughout follow-up. Our analysis did not find that baseline IIEF-EF scores predicted the extent of decline in sexual function over 5-year follow-up, although the number of subjects in the severe category was small. Interestingly, the IIEF-EF scores remained consistent throughout the study within each level of baseline IIEF-EF severity, and the different trend in this data compared with the data stratified by medical history of ED/EjD reinforces the importance of collecting patient reported outcomes along with medical history and diagnostic outcomes in future LUTS/BPH studies and when treating patients. This finding is consistent with the National Institute of Health Consensus Conference which established that evaluation of sexual dysfunction must include a detailed medical history and interview rather than exclusive reliance on written questionnaires.^28^

While the subjects with severe IIEF-EF scores at baseline appear to have some improvement in IIEF-EF during follow-up, this may be an artifact related to the small number of subjects in this group at 5 years (N = 3). Ejaculatory function based on the MSHQ-EjD function scores declined and reached statistical significance from 3 to 5 years, yet there was no corresponding significant change in the MSHQ-EjD bother scores at 5 years. The stability in the MSHQ-EjD bother score suggests a lack of clinically significant decline, although prior studies have reported that a significant portion of men are not bothered by symptoms of ED and/or EjD.^22^^,^^29^

This study is not without limitations. First, while the data were collected as part of a randomized, controlled study, the analysis presented here is part of a post-hoc analysis and the study was not powered to differentiate between ED/EjD subgroups. We chose not to use any method to impute missing data since using last observation carried forward, for example, could dampen the impact of aging on scores which was important to our analysis. Further, there was attrition during long-term follow-up since men who took medication or received other treatment during follow-up that could have favorably biased the outcomes attributed to Rezum were removed from follow-up. For this reason, there are some timepoints in long-term follow-up with a small number of observations.

Despite these limitations, the study provides important insight for clinicians who are advising patients on treatment options for LUTS/BPH. While sexual function after medical and surgical treatments are of clinical concern, the results of this study suggest that patients can anticipate durable improvement in LUTS/BPH symptoms for at least 5 years after a single treatment with water vapor thermal therapy with no apparent impact on sexual function. Further, the slight decline in sexual function in this population is aligned with published data on the normal para-aging process and based on the delayed timeframe of the gradual decline, does not suggest any relationship to water vapor thermal therapy for LUTS/BPH.

## CONCLUSIONS

The study results are consistent with the prior 3- and 4-year follow-up data and confirm that water vapor thermal therapy provides durable improvement in LUTS through 5-year follow-up with no clinically relevant impact on ED and EjD in this aging population.

## STATEMENT OF AUTHORSHIP

Kevin T. McVary: Conceptualization, Methodology, Investigation, Writing – Original Draft, Writing – Review & Editing, Funding Acquisition, Supervision; Ahmad El-Arabi: Methodology, Writing – Original Draft, Writing – Review & Editing; Claus Roehrborn: Conceptualization, Methodology, Investigation, Writing – Original Draft, Writing – Review & Editing, Supervision.

## Appendix Supplementary materials

Image, application 1

## Footnotes

Supplementary material associated with this article can be found in the online version at doi:10.1016/j.esxm.2021.100454.
