# Supplementary material for: Preservation of Sexual Function 5 Years After Water Vapor Thermal Therapy for Benign Prostatic Hyperplasia
Source: Sex Med. 2021 Oct 30;9(6):100454. doi: 10.1016/j.esxm.2021.100454 (PMC8766265; doi:10.1016/j.esxm.2021.100454)
Supplement: Supplementary file 1 [file mmc1.docx]

## Supplement Table. Paired Outcome Measures after Water Vapor Thermal Therapy in Sexually Active Subgroup, Baseline through 5 Years

|  | **Baseline** | **3 Months** | **6 Months** | **1 Year** | **2 Years** | **3 Years** | **4 Years** | **5 Years** |
| --- | --- | --- | --- | --- | --- | --- | --- | --- |
| **IPSS*** | | | | | | | | |
| No. (paired values) | 125 | 122 | 117 | 108 | 92 | 81 | 73 | 67 |
| Mean ± SD baseline | 21.9 ± 5.4 | 21.9 ± 5.4 | 22.0 ± 5.4 | 21.7 ± 5.5 | 21.1 ± 5.0 | 21.1 ± 5.1 | 21.2 ± 4.8 | 21.2 ± 5.0 |
| Mean ± SD follow-up |  | 10.2 ± 6.2 | 9.8 ± 6.5 | 9.6 ± 6.5 | 10.3 ± 6.1 | 9.0 ± 4.8 | 10.5 ± 7.0 | 10.4 ± 7.1 |
| Change ± SD |  | -11.7 ± 7.4 | -12.2 ± 7.6 | -12.1 ± 7.4 | -10.8 ± 7.9 | -12.1 ± 6.9 | -10.7 ± 7.8 | -10.8 ± 8.1 |
| % Change ± SD |  | -51.2 ± 31.4 | -53.9 ± 30.0 | -54.1 ± 30.0 | -47.2 ± 36.8 | -54.3 ± 27.1 | -46.1 ± 47.5 | -45.9 ± 48.4 |
| P Value^1^ |  | <.0001 | <.0001 | <.0001 | <.0001 | <.0001 | <.0001 | <.0001 |
| **IPSS QoL*** | | | | | | | | |
| No. (paired values) | 125 | 122 | 117 | 108 | 92 | 81 | 73 | 67 |
| Mean ± SD baseline | 4.2 ± 1.2 | 4.2 ± 1.2 | 4.2 ± 1.2 | 4.1 ± 1.2 | 4.1 ± 1.1 | 4.0 ± 1.1 | 4.1 ± 1.1 | 4.0 ± 1.1 |
| Mean ± SD follow-up |  | 2.1 ± 1.4 | 1.9 ± 1.5 | 1.9 ± 1.3 | 2.0 ± 1.3 | 1.7 ± 1.1 | 2.2 ± 1.4 | 2.0 ± 1.3 |
| Change ± SD |  | -2.1 ± 1.6 | -2.3 ± 1.6 | -2.3 ± 1.5 | -2.1 ± 1.6 | -2.3 ± 1.5 | -1.9 ± 1.6 | -2.0 ± 1.6 |
| % Change ± SD |  | -46.2 ± 37.9 | -53.5 ± 35.5 | -53.6 ± 33.8 | -49.0 ± 37.3 | -54.3 ± 31.3 | -44.3 ± 37.8 | -47.8 ± 34.7 |
| P Value^1^ |  | <.0001 | <.0001 | <.0001 | <.0001 | <.0001 | <.0001 | <.0001 |
| **Qmax***** | | | | | | | | |
| No. (paired values) | 125 | 121 | 113 | 107 | 91 | 80 | 72 | 65 |
| Mean ± SD baseline | 10.0 ± 2.8 | 10.0 ± 2.8 | 9.9 ± 2.8 | 10.0 ± 2.8 | 10.1 ± 2.9 | 10.0 ± 2.9 | 9.9 ± 2.9 | 10.0 ± 3.0 |
| Mean ± SD follow-up |  | 16.6 ± 8.0 | 15.7 ± 6.6 | 15.7 ± 7.2 | 14.3 ± 6.7 | 12.9 ± 5.6 | 13.0 ± 5.6 | 13.9 ± 5.8 |
| Change ± SD |  | 6.6 ± 7.8 | 5.8 ± 6.6 | 5.7 ± 7.2 | 4.2 ± 7.1 | 2.9 ± 5.5 | 3.1 ± 5.9 | 3.9 ± 6.2 |
| % Change ± SD |  | 72.3 ± 88.0 | 67.6 ± 83.8 | 64.2 ± 78.6 | 50.3 ± 80.5 | 34.1 ± 63.0 | 39.0 ± 68.0 | 47.7 ± 72.1 |
| P Value^1^ |  | <.0001 | <.0001 | <.0001 | <.0001 | <.0001 | <.0001 | <.0001 |
| **BPHII*** | | | | | | | | |
| No. (paired values) | 125 | 122 | 117 | 108 | 92 | 81 | 73 | 67 |
| Mean ± SD baseline | 5.9 ± 3.0 | 5.9 ± 2.9 | 5.9 ± 2.9 | 5.7 ± 2.8 | 5.4 ± 2.8 | 5.3 ± 2.9 | 5.3 ± 2.9 | 5.3 ± 2.9 |
| Mean ± SD follow-up |  | 2.7 ± 2.7 | 2.1 ± 2.5 | 1.7 ± 2.3 | 2.1 ± 2.4 | 1.7 ± 2.2 | 2.0 ± 2.6 | 2.2 ± 2.8 |
| Change ± SD |  | -3.2 ± 3.5 | -3.7 ± 3.1 | -3.9 ± 3.2 | -3.3 ± 3.2 | -3.7 ± 3.5 | -3.3 ± 3.7 | -3.1 ± 3.7 |
| % Change ± SD |  | -40.7 ± 89.2 | -62.5 ± 50.1 | -64.3 ± 51.1 | -55.2 ± 49.8 | -60.0 ± 70.0 | -52.1 ± 68.8 | -44.3 ± 85.3 |
| P Value^1^ |  | <.0001 | <.0001 | <.0001 | <.0001 | <.0001 | <.0001 | <.0001 |
| **IIEF-EF***** | | | | | | | | |
| No. (paired values) | 125 | 119 | 112 | 103 | 89 | 78 | 71 | 65 |
| Mean ± SD baseline | 22.6 ± 7.4 | 22.8 ± 7.3 | 22.7 ± 7.4 | 23.2 ± 6.8 | 22.9 ± 7.0 | 23.1 ± 7.2 | 23.6 ± 6.7 | 23.8 ± 6.8 |
| Mean ± SD follow-up |  | 22.4 ± 8.8 | 22.2 ± 8.9 | 21.9 ± 8.9 | 21.6 ± 8.7 | 21.3 ± 9.4 | 21.1 ± 9.8 | 21.0 ± 10.2 |
| Change ± SD |  | -0.4 ± 7.8 | -0.4 ± 6.6 | -1.3 ± 7.8 | -1.3 ± 7.6 | -1.9 ± 8.4 | -2.5 ± 8.8 | -2.8 ± 9.3 |
| % Change ± SD |  | 3.7 ± 44.8 | 1.5 ± 40.5 | -1.6 ± 44.5 | -1.8 ± 44.5 | -4.0 ± 47.0 | -8.5 ± 49.3 | -10.0 ± 47.9 |
| P Value^1^ |  | 0.5510 | 0.5029 | 0.1054 | 0.1017 | 0.0500 | 0.0170 | 0.0158 |
| **MSHQ – Function**** | | | | | | | | |
| No. (paired values) | 125 | 120 | 111 | 104 | 88 | 79 | 69 | 63 |
| Mean ± SD baseline | 9.4 ± 3.3 | 9.5 ± 3.3 | 9.6 ± 3.2 | 9.6 ± 3.2 | 9.8 ± 3.1 | 9.9 ± 3.2 | 10.1 ± 3.0 | 10.2 ± 3.0 |
| Mean ± SD follow-up |  | 9.7 ± 4.6 | 9.4 ± 4.2 | 9.2 ± 4.1 | 9.1 ± 4.4 | 8.6 ± 4.5 | 8.5 ± 4.5 | 8.4 ± 4.5 |
| Change ± SD |  | 0.2 ± 4.2 | -0.2 ± 3.7 | -0.4 ± 3.5 | -0.7 ± 4.0 | -1.4 ± 3.7 | -1.6 ± 4.3 | -1.9 ± 3.9 |
| % Change ± SD |  | 8.8 ± 59.9 | 2.6 ± 48.2 | 3.1 ± 66.7 | 1.8 ± 74.8 | -12.8 ± 46.8 | -11.6 ± 52.8 | -16.2 ± 46.0 |
| P Value^1^ |  | 0.6006 | 0.5398 | 0.2332 | 0.1266 | 0.0019 | 0.0025 | 0.0004 |
| **MSHQ – Bother*** | | | | | | | | |
| No. (paired values) | 125 | 120 | 114 | 105 | 89 | 79 | 69 | 63 |
| Mean ± SD baseline | 2.1 ± 1.7 | 2.0 ± 1.7 | 2.1 ± 1.7 | 2.1 ± 1.6 | 2.1 ± 1.6 | 2.0 ± 1.7 | 2.0 ± 1.6 | 1.9 ± 1.6 |
| Mean ± SD follow-up |  | 1.8 ± 1.7 | 1.8 ± 1.6 | 1.7 ± 1.6 | 1.7 ± 1.7 | 1.6 ± 1.6 | 1.9 ± 1.7 | 1.6 ± 1.7 |
| Change ± SD |  | -0.3 ± 1.9 | -0.3 ± 1.9 | -0.4 ± 1.8 | -0.4 ± 1.8 | -0.4 ± 1.9 | -0.1 ± 1.9 | -0.3 ± 2.1 |
| % Change ± SD |  | -18.4 ± 76.2 | -9.9 ± 84.6 | -14.3 ± 90.2 | -23.4 ± 82.1 | -23.9 ± 84.6 | -15.0 ± 86.8 | -23.0 ± 95.1 |
| P Value^1^ |  | 0.1198 | 0.1099 | 0.0253 | 0.0290 | 0.0508 | 0.5626 | 0.1940 |
| BPHII: Benign Prostatic Hyperplasia Impact Index, EF: Erectile Function, EjD: Ejaculatory Dysfunction, IIEF: International Index of Erectile Function, IPSS: International Prostate Symptom Score, MSHQ: Male Sexual Health Questionnaire, QoL: Quality of Life, Qmax: Peak Flow Rate  ^1^ P-value is for the one-sample t-test of whether the paired change from baseline is different from zero.  Analysis population includes all treatment and crossover arm subjects that underwent treatment with Rezum, were sexually active at baseline, and did not have other medical treatments during the study period.  Percent change analyses do not include subjects with baseline values of zero.  * Decrease indicates improvement.  ** Decrease indicates a decline in function.  *** Increase indicates improvement. | | | | | | | | |
